# Polyethylene glycol-based colloidal electrode via water competition for ultra-stable aqueous Zn-I batteries

**DOI:** 10.1016/j.isci.2024.111229

**Published:** 2024-10-22

**Authors:** Kaiqiang Zhang, Chao Wu, Luoya Wang, Changlong Ma, Jilei Ye, Yuping Wu

**Affiliations:** 1 School of Energy Sciences and Engineering, Nanjing Tech University, Nanjing, Jiangsu Province 211816, China

**Keywords:** electrochemical energy storage, electrochemistry, materials science

## Abstract

Current solid- and liquid-state electrode materials with extreme physical states show inherent limitation in achieving the ultra-stable batteries. Herein, we present a colloidal electrode design with an intermediate physical state to integrate the advantages of both solid- and liquid-state materials. The colloidal electrode was designed based on the inherent water competition effect of (SO_4_)^2−^ from the aqueous electrolyte and inherently water-soluble polyethylene glycol (PEG)/ZnI_2_ from the cathode. The constructed aqueous Zn||PEG/ZnI_2_ colloid battery demonstrated ultra-stable cycling performance with Coulombic efficiencies approaching 100% and a capacity retention of 86.7% over 10,700 cycles, without requiring anodic modification. In addition, the battery also exhibits compatibility with multiple operating conditions including fluctuating charging, limited self-discharging rate, different charging statuses, and fast charging. Moreover, the battery also shows practical potential by integrating with a photovoltaic solar panel charging. This design provides a broad platform for building the next-generation aqueous batteries with ultra-long lifetime.

## Highlights

• We present a soft colloidal electrode design with an intermediate physical state
• The colloidal electrode was designed through the inherent water competition effect
• The constructed aqueous battery demonstrated ultra-stable cycling performance

## Graphical abstract

**Figure:**
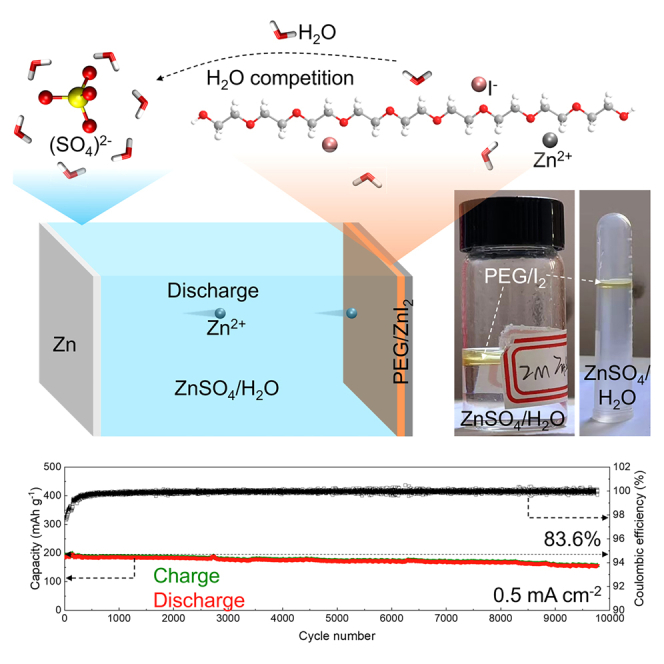

## Introduction

The development of electrode materials is crucial for creating ultra-stable cycling performance of batteries that can reduce daily costs.^1^^,^^2^^,^^3^^,^^4^^,^^5^^,^^6^ Currently, the efficient lifespan of lithium-ion batteries is approximately 2,000 cycles, or around 10 years, with a capacity retention ratio of about 80%.^7^^,^^8^ In contrast, aqueous batteries, known for their reliable safety and low costs, typically have a more limited cycling lifespan.^9^^,^^10^^,^^11^^,^^12^ To facilitate the development of ultra-long-lifetime batteries, we analyzed current battery construction characteristics and proposed our innovative solution.

Current battery constructions include various configurations such as liquid||liquid, solid||liquid||solid, solid||solid||solid, liquid||liquid||liquid, and solid||liquid||liquid structures.^13^^,^^14^^,^^15^^,^^16^^,^^17^^,^^18^ Typical prototypes encompass redox-flow batteries, metal-ion batteries, all-solid-state batteries, molten salt batteries, and liquid-electrode batteries.^19^^,^^20^^,^^21^^,^^22^^,^^23^^,^^24^^,^^25^^,^^26^^,^^27^^,^^28^ These constructions rely on solid and liquid materials with extreme physical states. The charge storage process in batteries is determined by the accommodation ability of charge carriers in electrode materials and the shuttling ability of charge carriers in electrolytes, which must tolerate repeated atomic stress within solid-state materials.^29^ This inherently limits the battery lifespan. Liquid-state materials, while promising for charge carriers shuttling, face challenges with uncontrolled species migration in liquid-state electrodes.^30^ Overall, conventional battery materials offer advantages such as species fixation in solid-stage electrode materials and the absence of rigid atomic structure in liquid-electrode materials, but they also suffer from the disadvantages of each other’s strengths. These practical conditions suggest that an effective combination of solid- and liquid-state materials could be a promising solution for developing next-generation ultra-stable batteries.

**Figure 1:**
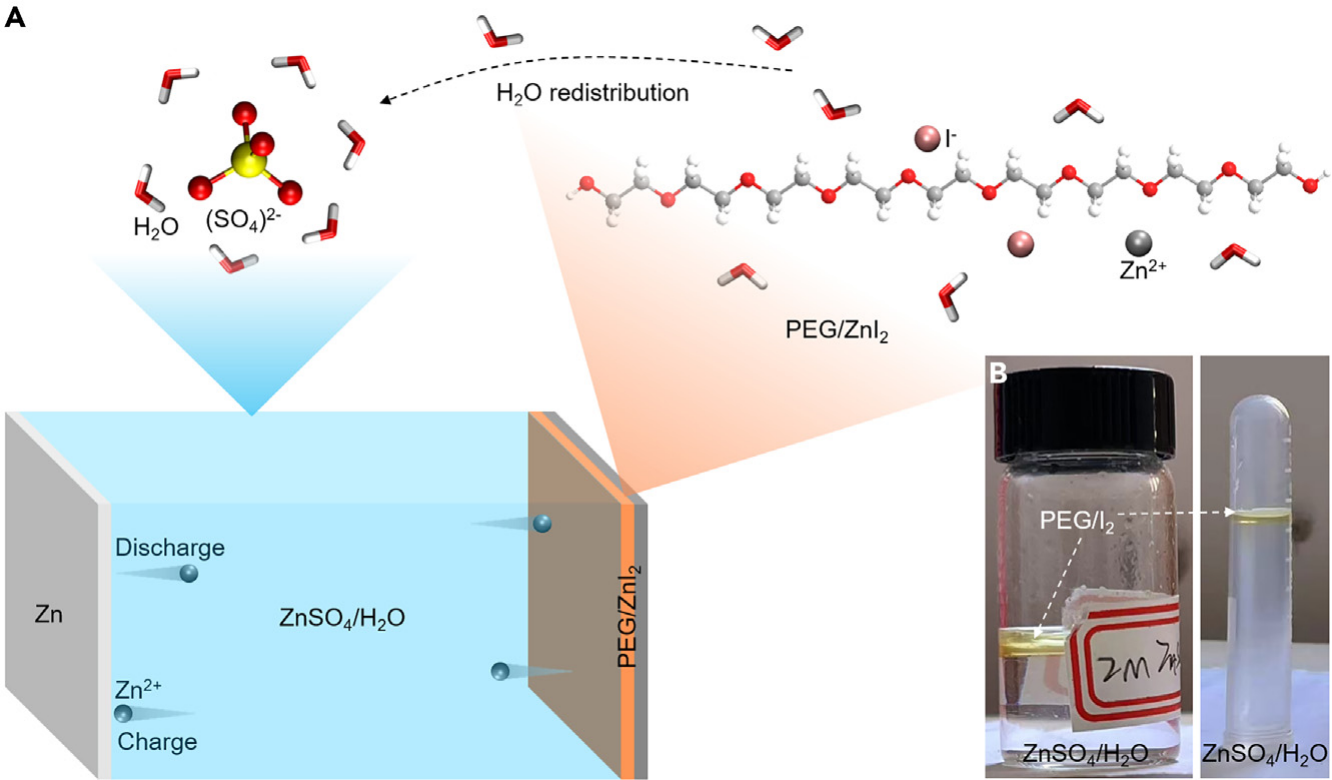
Schematic illustration of the construction of the aqueous Zn||PEG/ZnI_2_ colloid battery (A) Illustration of the water competition effect between (SO_4_)^2−^ and PEG. (B) Optical images of the insoluble PEG in 2 M ZnSO_4_ aqueous solution.

Based on our theoretical analysis of current battery constructions, we proposed and designed colloidal electrode materials with an intermediate physical state, rather than extreme solid or liquid states. This approach aims to combine the advantages of both solid- and liquid-state materials while avoiding their respective disadvantages. The non-extreme physical states refer to materials whose physical states are intermediate between solids and liquids. Colloidal electrode materials offer competitive fixation properties for redox-active species compared to conventional solid-state electrodes, while preventing the particle cracking or pulverization observed in conventional solid-state electrode materials, such as inorganic and organic particles. This approach, in principle, promises prolonged cycling stability. The soft, colloidal electrode material was realized through an inherent water competition effect between the (SO_4_)^2–^ species from the aqueous electrolyte and inherently water-soluble polyethylene glycol (PEG)/ZnI_2_ from the cathode, forming an aqueous Zn||PEG/ZnI_2_ colloid battery (Figure 1A). The colloidal electrode, devoid of a rigid lattice structure, effectively avoids lattice fatigue during repeated battery cycles and secures active species, thereby preventing capacity loss caused by the migration of redox-active species, such as iodide shuttling in aqueous Zn-I batteries (Figure 1B).^31^ Electrochemical performance demonstrated an ultra-long battery cycling lifespan exceeding 10,700 cycles. Furthermore, the aqueous Zn||PEG/ZnI_2_ colloid battery showed compatibility with various operating conditions, including fluctuating charging, limited self-discharging, different charging statuses, and fast-charging properties. Additionally, we demonstrated the integrity of the battery by charging it with a photovoltaic solar panel under sunlight, indicating the potential for practical applications. This battery design provides a broad platform for developing next-generation ultra-stable battery chemistries.

## Results and discussion

### Water competition effect demonstration

The water competition effect between (SO_4_)^2–^ ions and PEG species was investigated by dissolving PEG polymer in a 2 M ZnSO_4_ aqueous solution. To track the migration of PEG molecules, we colored them using yellow-colored iodine species. The inherently water-soluble PEG formed a separate layer on the surface of the 2 M ZnSO_4_ aqueous solution (Figures 1B, S1A, and Video S1). In contrast, PEG is fully soluble in deionized water (Figure S1B and Video S2). These distinct observations indicate that the presence of ZnSO_4_ inhibits the dissolution of PEG by reducing its interactions with water molecules that occur during the normal dissolution process. This difference in solubility suggests a water competition effect between ZnSO_4_ and PEG, where ZnSO_4_ controls the water content within PEG, resulting in limited PEG dissociation and the formation of a separate PEG colloid layer. This dual-layer structure meets the basic requirement for battery construction, providing immiscible electrolyte and cathode portions. Additionally, the iodide species are effectively retained within the PEG matrix, favoring stable battery cycling performance (Figure S1C).

Video S1. PEG/iodine/H2O in a 2 M ZnSO4 aqueous solution, related to Figure 1

Video S2. PEG/H2O in deionized water, related to Figure 1

To further confirm the origin of the water competition effect between ZnSO_4_ and PEG, we added ZnI_2_ to the PEG/H_2_O mixture. The ZnI_2_ fully dissolved, forming a transparent, colloid-like solution, indicating that the water competition effect is primarily determined by (SO_4_)^2–^ anions rather than Zn^2+^ cations. The unique property supports the battery’s effective operation by allowing only Zn^2+^ cations to shuttle between the 2 M ZnSO_4_ aqueous electrolyte and the PEG colloid cathode for charge balancing during battery charging and discharging (Figure S2). Additionally, the limited water molecules within the PEG colloid further facilitate Zn^2+^ migration (Figure S2), while the redox-active iodine species were efficiently encapsulated in the PEG colloid. Therefore, we designed the aqueous Zn||PEG/ZnI_2_ colloid battery using this inherent water competition effect between (SO_4_)^2−^ from the aqueous electrolyte and PEG from the cathode for ultra-stable aqueous Zn-I batteries.

### Electrochemical performance

**Figure 2:**
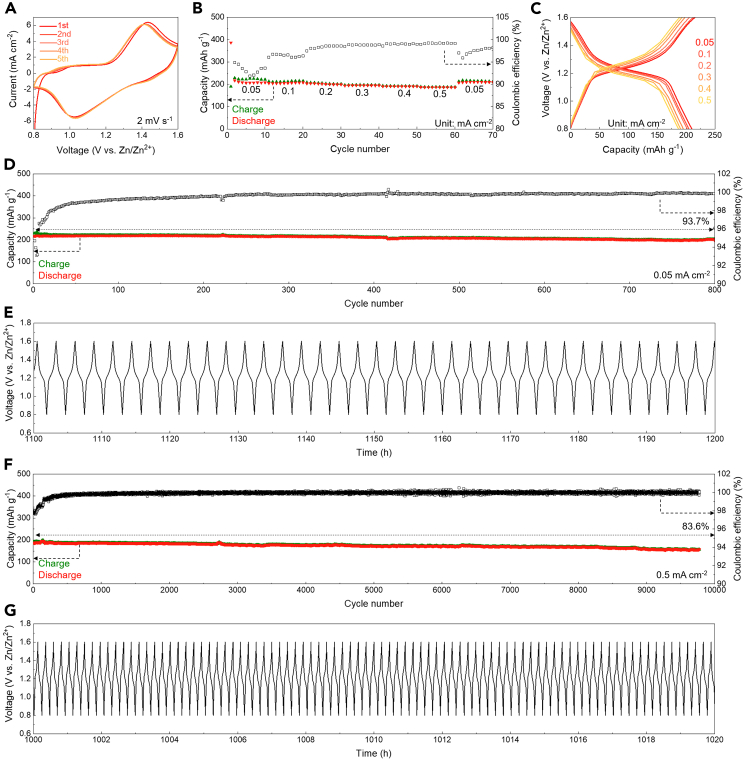
Electrochemical performance of the aqueous Zn||PEG/ZnI_2_ colloid battery (A) CV curve of the battery. (B and C) Rate performance of the battery. (D–G) Specific capacities and Coulombic efficiencies (D and F) and voltage profiles (E and G) of the battery.

The electrochemical performance of the aqueous Zn||PEG/ZnI_2_ colloid battery was thoroughly evaluated. Cyclic voltammetry (CV) curves, scanned at 2 mV s^−1^ by controlling the voltage variation, exhibited a pair of redox peaks at 1.41 (oxidative) and 1.03 (reductive) V vs. Zn/Zn^2+^, confirming the typical Zn-I redox chemistry, with the typical redox pairs of Zn/Zn^2+^ and I/I^−^ (Figure 2A).^32^ Additionally, the potential PEG molecular rearrangement was observed during battery operation. During the charging process of the aqueous Zn||PEG/ZnI_2_ colloid battery, PEG molecules responded to the applied electric field, as indicated by the increasing capacitance in the redox-inactive region (Figure S3A). Specifically, the CV curve of the battery showed capacitance-type current in the charging voltage range of 0.9–1.2 V vs. Zn/Zn^2+^ (Figure S3B) and in the discharging voltage range of 1.5–1.3 V vs. Zn/Zn^2+^ (Figure S3C). This capacitance-type response current was opposite to the normal redox peaks, where oxidative peaks occur at higher voltages and reductive peaks at lower voltages (Figure S3). This response current in the redox-inactive region indicated a capacitance-type charge storage process of PEG molecules in the cathode. This capacitance behavior is triggered after a short voltage increase, from 0.85 to 0.9 V vs. Zn/Zn^2+^ (Figures 2A and S3B), suggesting a potential PEG molecular rearrangement process driven by the applied electric field, which reverses during discharging (Figure S3D). This rearrangement explains why the capacitance behavior indicator is positioned opposite to the normal redox peaks. Further calculations of the electrostatic potential of the PEG molecular segments showed that O sites had concentrated electronegativity around −24 kcal mol^−1^, while the –CH_2_– site showed slight electro-positivity around 6 kcal mol^−1^, suggesting the presence of dipoles responsive to the applied electric field (Figure S3E). The polymeric PEG chains form a matrix with localized electrostatic fields that can host ions during battery charging and discharging.

Rate performance of the aqueous Zn||PEG/ZnI_2_ colloid battery was tested under various current densities of 0.05, 0.1, 0.2, 0.3, 0.4, and 0.5 mA cm^−2^. The battery demonstrated an 87.4% capacity retention after a 10-fold increase in operating current density, with voltage profiles showing consistent redox plateaus (Figures 2B and 2C). The lower Coulombic efficiency observed in the initial stages of the rate performance tests can be attributed to battery’s activation process (Figure 2B), which often results in a temporary decrease in efficiency. This activation effect is more pronounced when the battery is cycled at lower current densities (Figure 2D). The electrochemical kinetics was further analyzed using redox peaks polarization of CV curves and electrochemical impedance spectroscopy (EIS). The anodic and cathodic peaks exhibited voltage polarizations of 0.14 and 0.12 V, respectively (Figure S4A). Additionally, the EIS spectrum of the battery showed a charge transfer impedance of approximately 30 Ohms and an internal resistance of less than 5 Ohms (Figure S4B), indicating normal battery impedance.

Long-term galvanostatic charging and discharging tests of the aqueous Zn||PEG/ZnI_2_ colloid battery were conducted at 0.05 and 0.5 mA cm^−2^. After the initial battery activation and molecular rearrangement, the battery exhibited stable cycling performance over 800 and 9,700 cycles, with Coulombic efficiencies approaching 100% and capacity retentions of 93.7% and 83.6% at 0.05 and 0.5 mA cm^−2^, respectively (Figures 2D and 2F). The batteries showed consistent voltage profiles, confirming stable cycling performance and promising a long battery lifetime (Figures 2E and 2G). Long-term cycling performance of the battery was further confirmed by repeated testing. When cycling the battery at 0.5 mA cm^−2^, it continued to exhibit Coulombic efficiencies approaching 100% and a capacity retention of 77.8% over 10,000 cycles (Figure S5A). Furthermore, the battery maintained consistent voltage profiles throughout the entire cycling test process (Figure S5B). Additionally, the battery cycling performance has been demonstrated with a 5-fold increase in the iodide loading amount, showing stable cycling results (Figure S6). After a thorough evaluation and comparison with other research efforts, our work demonstrates comparable or even superior electrochemical performance (Table S1). Note that the PEG/ZnI_2_ solution is vulnerable to oxidation when exposed to the ambient environment, causing the color to turn pale yellow. This oxidation may impact the battery’s initial activation process (Figures S7A–S7C).

### Electrochemical performance under multiple operating conditions

#### Fluctuating charging

**Figure 3:**
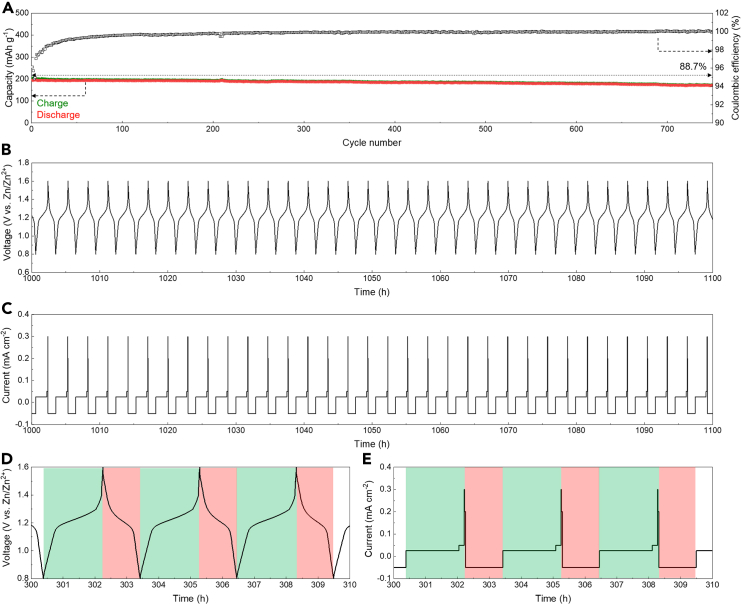
Fluctuating charging performance of the aqueous Zn||PEG/ZnI_2_ colloid battery (A) Specific capacities and Coulombic efficiencies during repeated cycling tests. (B–E) Corresponding voltage (B and D) and current (C and E) profiles of the battery during cycling tests.

Practical photovoltaic charging often faces the challenge of fluctuating power due to inconsistent sunlight. To demonstrate the compatibility of the aqueous Zn||PEG/ZnI_2_ colloid battery with such fluctuating charging conditions, we tested the batteries by charging them at fluctuating current densities of 0.025, 0.05, 0.3, and 0.2 mA cm^−2^, while discharging them at a consistent current density of 0.05 mA cm^−2^. As a result, the battery exhibited Coulombic efficiencies approaching 100% and an 88.7% capacity retention ratio over 750 cycles (Figure 3A). The operating process of the battery revealed consistent tracked voltage profiles in response to the current profiles, indicating stable battery operation (Figures 3B–3E). These results demonstrate the compatibility of the aqueous Zn||PEG/ZnI_2_ colloid battery with fluctuating charging behavior.

#### Battery self-discharging

**Figure 4:**
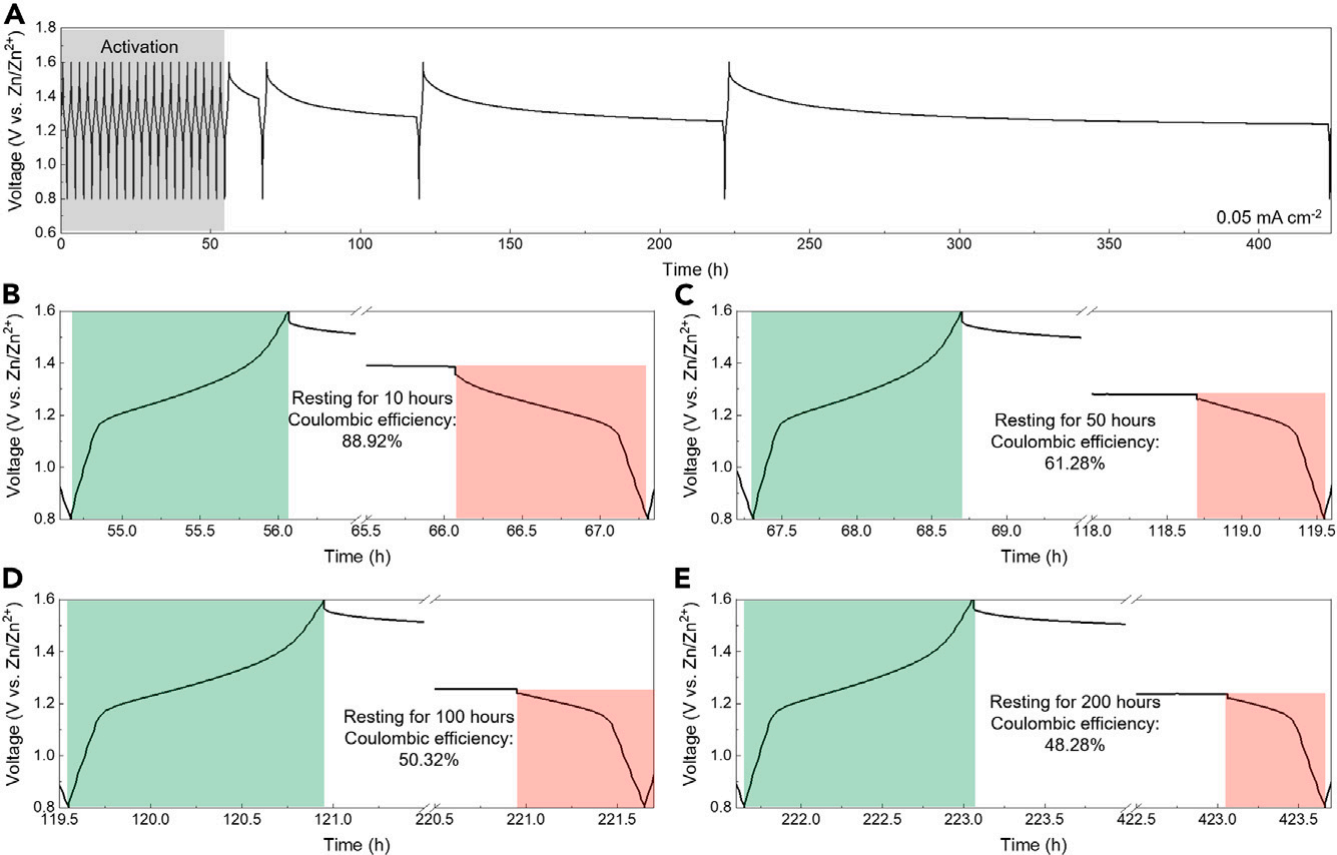
Self-discharging test of the aqueous Zn||PEG/ZnI_2_ colloid battery Overall (A) and individual voltage profiles (B–E) during the battery self-discharging tests.

Battery self-discharging is a significant factor for practical daily use. To measure the self-discharging rate of the aqueous Zn||PEG/ZnI_2_ colloid battery, we tested the battery by galvanostatically charging it at 0.05 mA cm^−2^ to 1.6 V vs. Zn/Zn^2+^, followed by resting for 10, 50, 100, and 200 h, respectively, and then discharging it directly (Figure 4A). The Coulombic efficiency parameter was used to evaluate the self-discharging rate. Prior to the resting periods, the battery underwent an initial activation treatment. The battery demonstrated Coulombic efficiencies of 88.92%, 61.28%, 50.32%, and 48.28% after resting for 10, 50, 100, and 200 h, respectively (Figures 4B–4E). Generally, the battery showed a gradual decrease in Coulombic efficiencies with increasing resting time. The increase in Coulombic efficiency observed in the initial stages of the self-discharging tests is likely due to the premature battery condition following the initial activation process after assembly. Overall, the Coulombic efficiency values after long-term resting periods, such as 200 h, provide a reliable measure of the battery’s self-discharging rate. Additionally, the charging capacity after each resting and discharging cycle remained nearly constant, demonstrating the effectiveness of the PEG/ZnI_2_ colloidal cathode in fixing the redox-active iodide species on the cathode side and preventing uncontrolled migration loss, thereby promoting an ultra-long battery lifespan.

#### Various charging statuses

**Figure 5:**
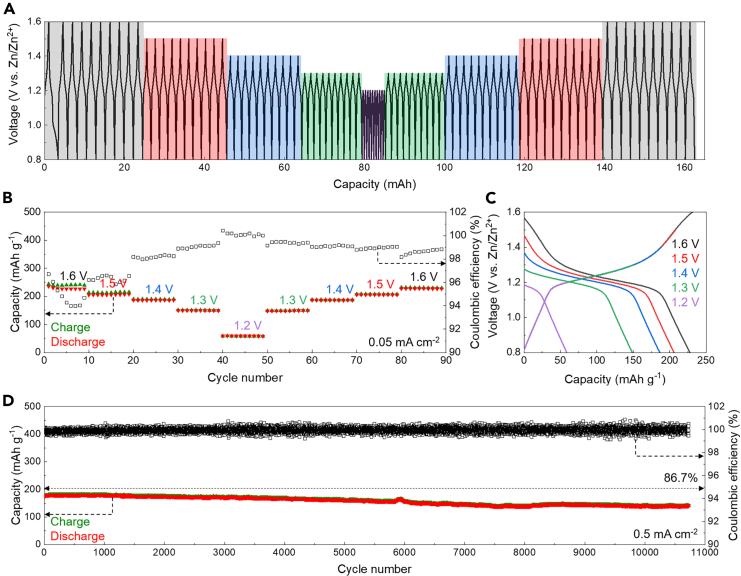
Compatibility of the aqueous Zn||PEG/ZnI_2_ colloid battery with various charging statuses Continuous voltage profiles (A), capacity and Coulombic efficiency values (B), representative voltage profiles for each charging status condition (C), and subsequent long-term cycling tests (D).

In daily use, batteries often start operating from various charging states rather than being fully charged. To demonstrate the compatibility of the aqueous Zn||PEG/ZnI_2_ colloid battery with such conditions, we tested the battery by galvanostatically charging it at 0.05 mA cm^−2^ to different cutoff voltages of 1.2, 1.3, 1.4, 1.5, and 1.6 V vs. Zn/Zn^2+^, followed by galvanostatic discharging at the same current density. The battery demonstrated Coulombic efficiencies approaching 100% and consistent voltage profiles (Figures 5A and 5B). Furthermore, the charging voltage profiles were consistent regardless of the charging cutoff voltages (Figure 5C), indicating the battery compatibility with different charging statuses. After the tests, the battery continued to operate stably, showing Coulombic efficiencies approaching 100% and 86.7% capacity retention after around 10,700 cycles (Figure 5D), indicating its long-term cycling efficiency. The battery demonstrated stable voltage profiles throughout the cycling tests (Figure S8), confirming its reliable cycling performance.

#### Fast-charging performance

**Figure 6:**
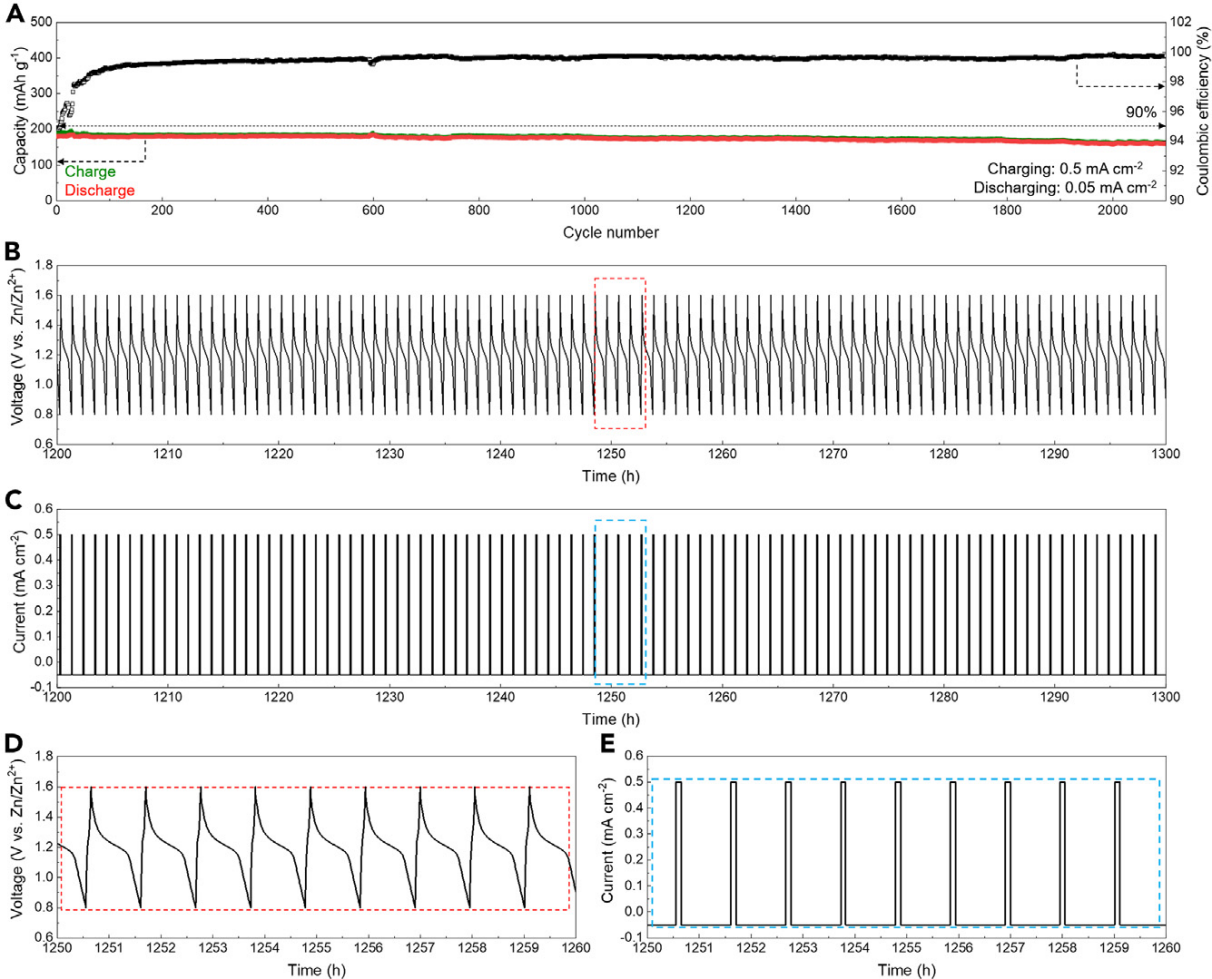
Fast-charging performance of the aqueous Zn||PEG/ZnI_2_ colloid battery (A) Specific capacity and Coulombic efficiency values of the battery. (B–E) Voltage (B and D) and current (C and E) profiles of the battery during fast-charging tests.

Fast-charging performance is crucial in current practical battery applications to improve charging efficiency.^33^ We demonstrated the fast-charging performance of the aqueous Zn||PEG/ZnI_2_ colloid battery by galvanostatically charging it at 0.5 mA cm^−2^ and discharging it at 0.05 mA cm^−2^. After the initial activation process, the battery delivered Coulombic efficiencies approaching 100% and a 90% capacity retention ratio over 2,100 cycles, with an 87.4% capacity retention during 10-fold fast charging compared to that charged at 0.05 mA cm^−2^ (Figure 6A). In response to the applied current densities, the battery exhibited consistent voltage profiles, indicating stably fast-charging capability (Figures 6B–6E).

#### Practical integration with photovoltaic solar panel charging

**Figure 7:**
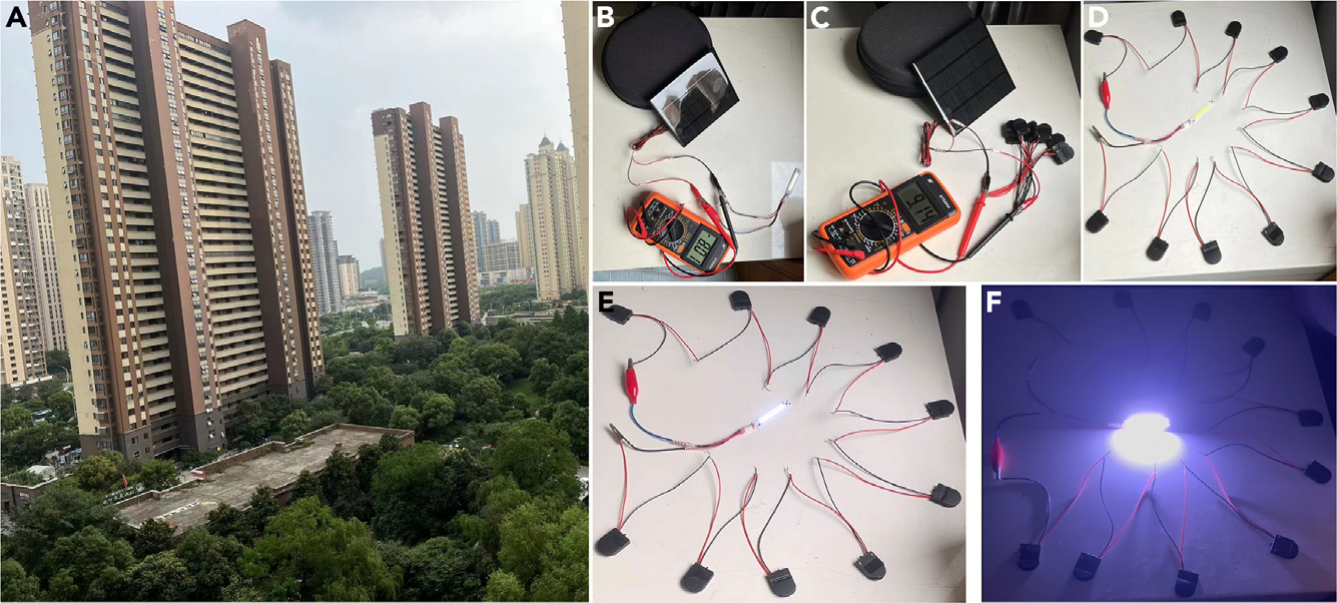
Practical integration demonstration of the aqueous Zn||PEG/ZnI_2_ colloid battery with photovoltaic solar panel charging (A) Local sunlight during the demonstration. (B) Using the photovoltaic solar panel with an 8 V output voltage to directly power a 10 V LED panel. (C) Using the photovoltaic solar panel with a 9.14 V output to charge the batteries in parallel. (D–F) Photovoltaic solar panel-charged 12 V LED panel during daytime (E) and at night (F).

The integration potential of the aqueous Zn||PEG/ZnI_2_ colloid battery with a practical photovoltaic solar panel was demonstrated by charging the batteries using a 10 V, 3 W, 300 mA photovoltaic solar panel under sunlight (Figure 7A). The photovoltaic solar panel exhibited an output voltage of approximately 8 V (Figure 7B). After adjusting the angle of the photovoltaic solar panel, the batteries connected in parallel were charged under a solar panel output voltage of around 9 V (Figure 7C). Following this practical photovoltaic solar panel charging, from 1 to 1.6 V vs. Zn/Zn^2+^ (Video S3), the charged aqueous Zn||PEG/ZnI_2_ colloid batteries were connected in series and used to power a 12 V, 1.5 W LED panel both during daytime and at night (Figures 7D–7F, Videos S4 and S5).

Video S3. Practical photovoltaic solar panel charging aqueous Zn||PEG/ZnI2 colloid battery, related to Figure 7

Video S4. Photovoltaic charged aqueous Zn||PEG/ZnI2 colloid battery powering a 12 V, 1.5 W LED panel during daytime, related to Figure 7

Video S5. Photovoltaic charged aqueous Zn||PEG/ZnI2 colloid battery powering a 12 V, 1.5 W LED panel at night, related to Figure 7

### Conclusion

In this article, we developed an aqueous Zn||PEG/ZnI_2_ colloid battery utilizing the inherent water molecular competition effect between (SO_4_)^2–^ ions from the electrolyte and PEG molecules from the cathode. The (SO_4_)^2–^ ions act as a molecular valve to limit the water content in PEG, thereby forming a colloidal state that combines the advantages of species fixation in solid-state electrode materials and the lack of rigid atomic structure in liquid-state electrode materials. The PEG/ZnI_2_ colloid, with its intermediate physical state, demonstrated ultra-stable cycling performance, achieving Coulombic efficiencies approaching 100% and a capacity retention of 86.7% over 10,700 cycles. Additionally, the battery exhibited compatibility with various operating conditions, including fluctuating charging, a limited self-discharging rate, different charging statuses, and fast-charging capability. Moreover, the battery demonstrated compatibility with practical photovoltaic solar panel charging conditions, suggesting its potential for large-scale static energy storage applications. The design concept of colloidal electrodes provides a broad platform and new perspective for developing next-generation ultra-stable battery chemistries.

### Limitations of the study

This study primarily focuses on the modification of cathode/electrolyte interface using the water competition effect. However, achieving balanced electrochemical performance typically requires modifications to both the anode/electrolyte and cathode/electrolyte interfaces. Additionally, our demonstrations were conducted using untreated zinc foils, which may limit the performance. Furthermore, the colloidal cathode is ionically conductive but lacks electronic conductivity, which constrains the overall kinetic performance of the battery.

## Resource availability

### Lead contact

Further information and any requests should be directed to and will be fulfilled by the lead contact, Kaiqiang Zhang (kaiqiangzhang@njtech.edu.cn).

### Materials availability

This study did not generate new unique reagents.

### Data and code availability

• All data generated or analyzed during this study are included in the manuscript and supplementary tables and figures.
• This paper does not report original code.
• Any additional information required to reanalyze the data reported in this paper is available from the lead contact upon request.

## Acknowledgments

The authors are grateful to the support by the 10.13039/501100001809National Natural Science Foundation of China (22109069) and the 10.13039/501100004608Natural Science Foundation of Jiangsu Province (BK20221446).

## Author contributions

Conceptualization, K.Z. and J.Y.; methodology, K.Z., J.Y., and Y.W.; investigation, K.Z., C.W., L.W., and C.M.; writing – original draft, K.Z.; writing – review and editing, K.Z., J.Y., and Y.W.; funding acquisition, K.Z. and J.Y.; resources, K.Z., J.Y., and Y.W.; supervision, K.Z. and J.Y.

## Declaration of interests

The authors declare no competing interests.

## STAR★Methods

### Key resources table

**Table:** 

REAGENT or RESOURCE	SOURCE	IDENTIFIER
**Chemicals, peptides, and recombinant proteins**		

ZnSO_4_	Sigma-Aldrich	CAS No.: 7446-19-7
Polyethylene glycol	Sigma-Aldrich	CAS No.: 25322-68-3
ZnI_2_	Sigma-Aldrich	CAS No.: 10139-47-6

**Software and algorithms**		

Gaussian 16 package	Gaussian, Inc.	https://gaussian.com/
Visual Molecular Dynamics (VMD)	University of Illinois at Urbana-Champaign (UIUC)	https://www.ks.uiuc.edu/Research/vmd/
Multiwfn 3.7 program	Beijing Kein Research Center for Natural Sciences	http://sobereva.com/multiwfn/

### Method details

#### Demonstration of water competition effect

Polyethylene glycol (PEG) (CAS No.: 25322-68-3) was purchased from Sigma**-**Aldrich. The PEG has a molecular range of 3500–4500. The PEG colloid was prepared by dissolving 1 g of PEG in 20 mL of deionized water with continuous stirring. This PEG colloid was colored using iodine for the subsequent water molecular competition demonstration with ZnSO_4_ (CAS No.: 7446-19-7). The selective water competition effect between the ZnSO_4_ aqueous electrolyte and PEG colloid was demonstrated by adding the colored PEG colloid to a 2 M ZnSO_4_ aqueous solution, followed by mixing. Ultimately, the PEG colloid separated from the 2 M ZnSO_4_ aqueous solution. The colored PEG colloid was also added to deionized water as a control experiment. All experiments were conducted at room temperature in an ambient environment. UV-Vis spectra of the ZnSO_4_/H_2_O solutions were collected on a Shimadzu UV-2456 spectrophotometer.

#### Electrolyte preparation

The 2 M ZnSO_4_ aqueous solution was prepared by dissolving ZnSO_4_ in deionized water for the subsequent battery assembly.

#### Preparation of current collector

An activated carbon slurry was prepared by dispersing activated carbon, carbon black, and polyvinylidene fluoride binder (in a weight ratio of 7:2:1) in N-methyl-2-pyrrolidone solvent, followed by constant stirring to form a uniform milky slurry. The current collector was then prepared by casting the slurry onto a carbon paper host and vacuum drying at 60°C for 8 h. The dried carbon paper was then cut into round discs with a diameter of 14 mm.

#### Preparation of PEG/ZnI_2_ colloid cathode

The PEG/ZnI_2_ colloid cathode was prepared by dissolving 1 g of PEG and 0.3 g of ZnI_2_ (CAS No.: 10139-47-6) in 20 mL of deionized water. The PEG/ZnI_2_ colloid was then loaded onto the as-prepared current collector with a loading amount of 25 μL cm^−2^, with iodide loading amount of approximately 0.4 mg cm^−2^, followed by drying with an air blower. The cathode preparation process was conducted in an ambient environment. The colloid cathode with a larger iodide loading amount was prepared by increasing the amount of ZnI_2_ 5-fold.

#### Battery assembly process

Coin–type aqueous Zn||PEG/ZnI_2_ colloid batteries were fabricated using Zn foil (50 μm in thickness) as the anode, 60 μL of 2 M ZnSO_4_ aqueous solution as the electrolyte, and the PEG/ZnI_2_ colloid as the cathode. The battery assembly process was conducted at room temperature in an ambient environment.

#### Electrochemical tests

Cyclic voltammetry (CV) curves of the aqueous Zn||PEG/ZnI_2_ colloid battery were scanned at 2, 4, 6, 8, and 10 mV s^−1^ within a voltage range of 0.8–1.6 V vs. Zn/Zn^2+^ using a CHI-760 (Chenhua) electrochemical workstation. Additionally, electrochemical impedance spectroscopy (EIS) of the battery was recorded under an amplitude of 5 mV in a frequency range of 10 mHz to 10^5^ Hz. Rate performance and continuous charging and discharging tests were performed using a LANHE tester. Specifically, the rate performance was recorded at current densities of 0.05, 0.1, 0.2, 0.3, 0.4, and 0.5 mA cm^−2^, while long-term continuous cycling tests were conducted at current densities of 0.05 and 0.5 mA cm^−2^. All capacity values were calculated based on the loaded amounts of iodide.

#### Multiple operating condition tests

The aqueous Zn||PEG/ZnI_2_ colloid battery was further tested under various operational conditions, including fluctuating charging current densities, self-discharging during resting, different charging cut-off voltages, and fast-charging performances. Fluctuating charging was tested by charging the battery at current densities of 0.025, 0.05, 0.3, and 0.2 mA cm^−2^ in a single charging process, followed by discharging at 0.05 mA cm^−2^. Self-discharging rate was measured by charging the battery at 0.05 mA cm^−2^ to 1.6 V vs. Zn/Zn^2+^, then resting for 10, 50, 100, 200, and 500 h, followed by direct discharging to 0.8 V vs. Zn/Zn^2+^. The battery was also tested by charging to 1.6, 1.5, 1.4, 1.3, and 1.2 V vs. Zn/Zn^2+^, followed by discharging to 0.8 V vs. Zn/Zn^2+^ at 0.05 mA cm^−2^. The fast-charging performance was evaluated by charging the battery at 0.5 mA cm^−2^ and discharging at 0.05 mA cm^−2^.

#### Integration ability with photovoltaic solar panel charging

The integration potential of the aqueous Zn||PEG/ZnI_2_ colloid battery with a photovoltaic solar panel was demonstrated by directly charging the batteries in parallel to 1.6 V vs. Zn/Zn^2+^ using a photovoltaic solar panel (10 V, 3 W, 300 mA) under local sunlight. The batteries were then connected in series to power an LED lamp (12 V, 1.5 W).

#### Theoretical calculations

The lowest-energy geometry for the PEG molecule was determined in gas phase using the Gaussian 16 package, employing tight convergence criteria. Calculations utilized the B3LYP hybrid functional (Becke 3-parameter exchange functional combined with Lee-Yang-Parr correlation functional) and the 6-31G (d, p) basis set for all atoms. The results of the molecular electrostatic potential were calculated using the Multiwfn 3.7 program, with.fch files converted and supplied by the Gaussian 16 package.^34^ Visualization of the molecular electrostatic potential plot was performed using Visual Molecule Dynamics software.^35^
