# Supplementary material for: Polyethylene glycol-based colloidal electrode via water competition for ultra-stable aqueous Zn-I batteries
Source: iScience. 2024 Oct 22;27(11):111229. doi: 10.1016/j.isci.2024.111229 (PMC11577285; doi:10.1016/j.isci.2024.111229)
Supplement: Document S1. Figures S1–S8 and Table S1 [file mmc1.pdf]

iScience, Volume *27*

Supplemental information

Polyethylene glycol-based colloidal electrode via water competition for ultra-stable aqueous Zn-I batteries

Kaiqiang Zhang, Chao Wu, Luoya Wang, Changlong Ma, Jilei Ye, and Yuping Wu

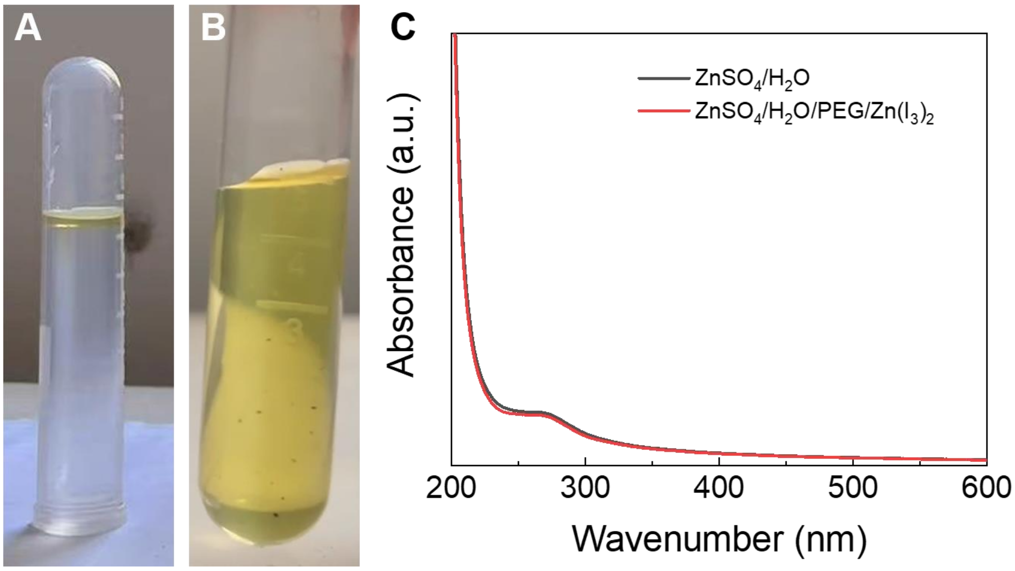

Figure S1. Demonstration of selective water distribution due to the competition effect between $(SO_4)^{2-}$ ions and PEG on water molecules, related to Figure 1.

Optical images of colored PEG colloid on (A) 2 M $ZnSO_4$ aqueous solution and (B) deionized water. (C) UV-Vis spectra of the 2 M $ZnSO_4$ aqueous before and after adding the iodide containing colloid.

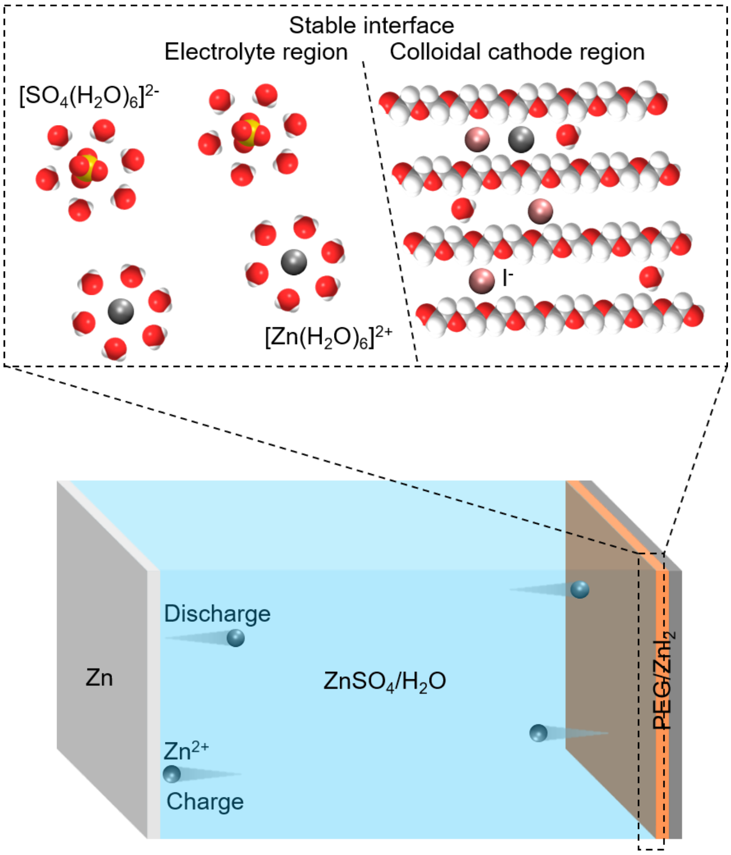

Figure S2. Illustration of the electrolyte/cathode interfacial structure and zinc ion migration in the PEG/ZnI_2 colloid cathode of the aqueous Zn||PEG/ZnI_2 colloid battery, related to Figure 1.

The arrangement of PEG molecules is depicted to demonstrate the molecular aggregation phenomenon.

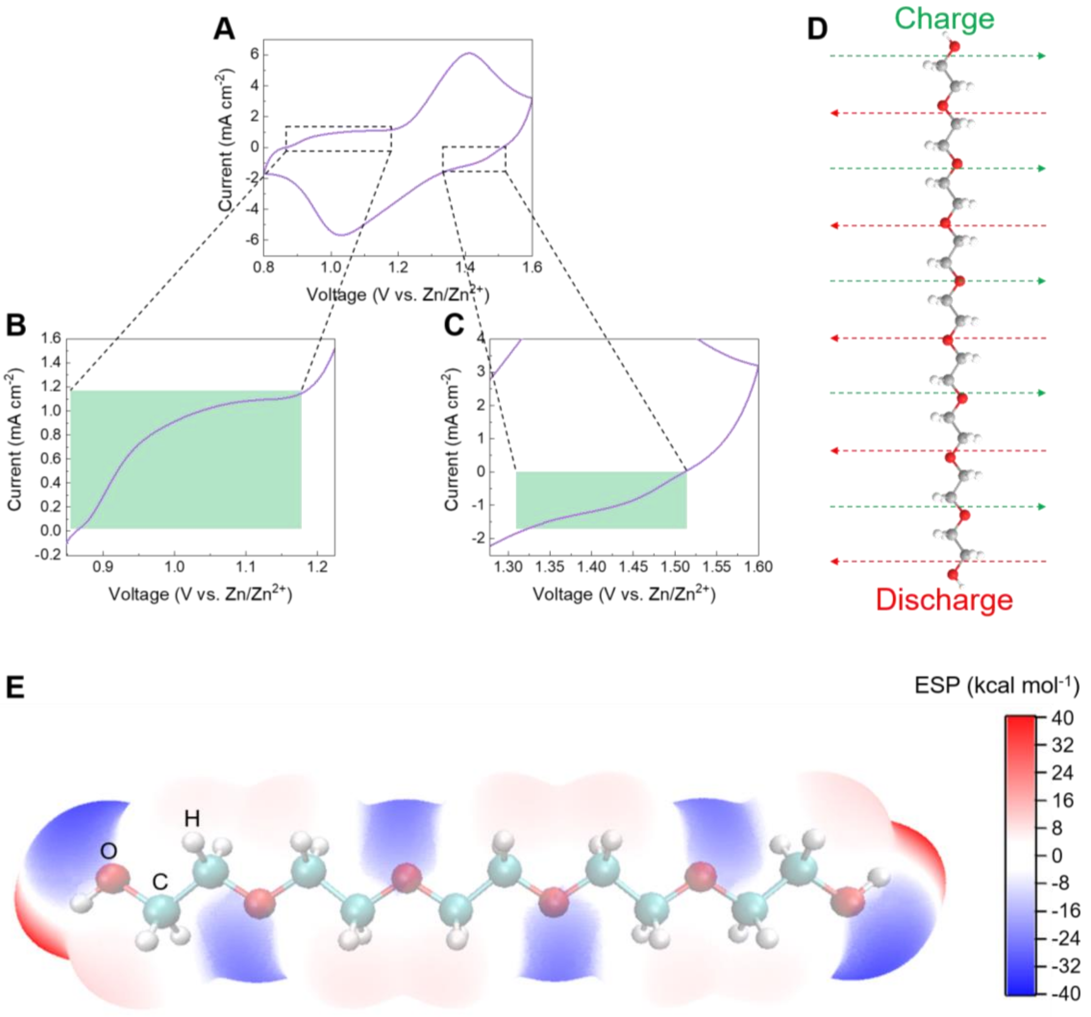

Figure S3. Analysis of PEG molecular rearrangement during the operation of the aqueous Zn||PEG/ZnI_2 colloid battery, related to Figure 2.

(A–C) Overall CV curve (A) and magnified oxidative (B), reductive (C) CV curve. (D) Applied electric field on PEG molecules during charging (green) and discharging (red). (E) Calculated electrostatic potential for the PEG molecular units.

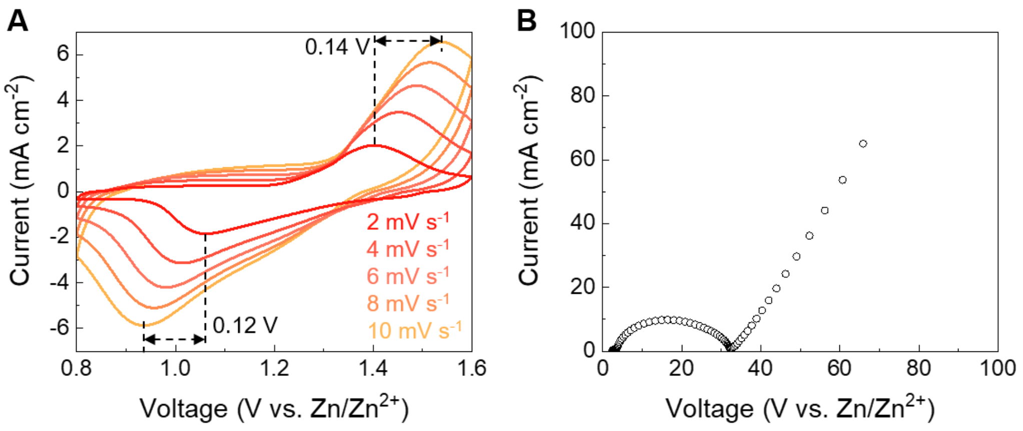

Figure S4. Electrochemical kinetics of the aqueous Zn||PEG/ZnI_2 colloid battery, related to Figure 2.

(A,B) Multiple CV curves scanned at 2, 4, 6, 8, and 10 mV s^{-1} (A) and EIS spectrum (B) of the aqueous Zn||PEG/ZnI_2 colloid battery.

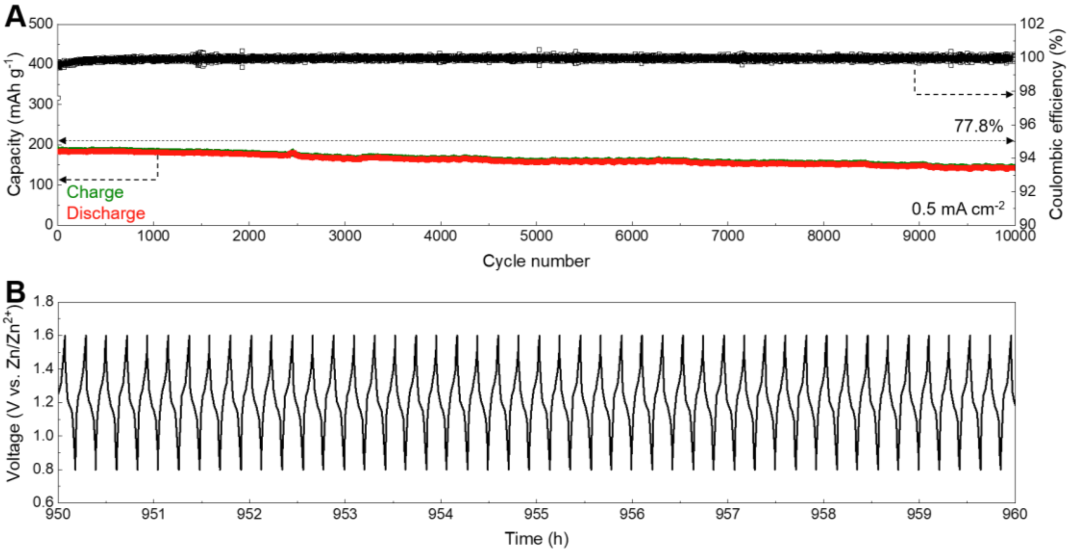

Figure S5. Repeated cycling tests for the aqueous Zn||PEG/ZnI$_2$ colloid battery, related to Figure 2.

(A–D) Capacity values (A) and overall (B), magnified (C,D) voltage profiles.

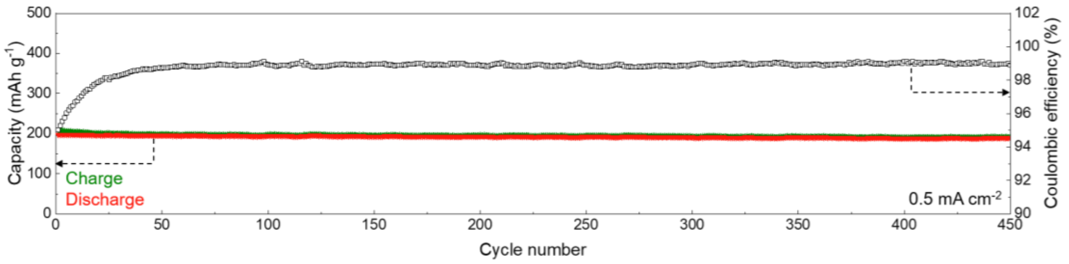

Figure S6. Repeated cycling tests for the aqueous Zn||PEG/ZnI$_2$ colloid battery with a larger iodide loading amount, related to Figure 2.

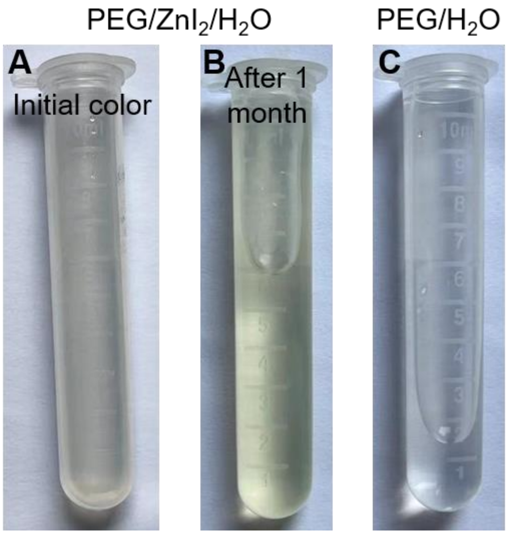

Figure S7. Optical images of different mixtures, related to Figure 2.

(A) Initial PEG/ZnI_2/H_2O, (B) PEG/ZnI_2/H_2O after one month, and (C) PEG/H_2O.

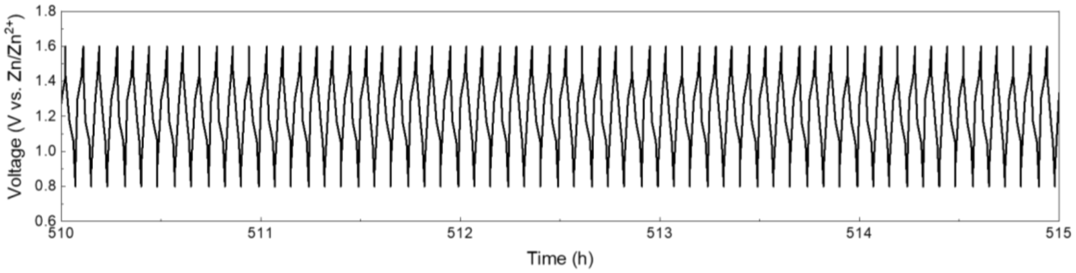

Figure S8. Representative voltage profiles of the aqueous Zn||PEG/ZnI$_2$ colloid battery, related to Figure 5.

Table S1. Comparison of the electrochemical performance of Zn-I batteries, related to Figure 2.

Concept	Current density	Cycling number	Capacity retention	Coulombic efficiency	Ref.
Colloidal electrode	0.5 mA cm^{-2}	10700	86.7%	~100%	This work
MOF membrane	1920 mA g^{-1}	6000	84.6%	99.65%	[S1]
Suspension electrolyte	20 C	40000	71.1%	100.1%	[S2]
Tin-based perovskite cathodes	1 A g^{-1}	5700	95%	~99%	[S3]
Graphene oxide and water-in-salt electrolyte	50 mA cm^{-2}	2000	>100%	~100%	[S4]
Cathode additive	20 C	10000	~80%	~100%	[S5]
Ionic liquid additive	4 A g^{-1}	18000	~78%	~100%	[S6]
Cathode design	10 mA cm^{-2}	12000	91%	~92%	[S7]
Functionalized graphene electrodes	5 A g^{-1}	2000	96.7%	~100%	[S8]
Molecular catalysis	10 A g^{-1}	62000	~75%	~100%	[S9]
Metal-nitrogen-carbon	2 A g^{-1}	10000	~88%	~100%	[S10]

References

S1. Yang, H., Qiao, Y., Chang, Z., Deng, H., He, P., and Zhou, H. (2020). A metal-organic framework as a multifunctional ionic sieve membrane for long-life aqueous zinc-iodide batteries. Adv. Mater. *32*, 2004240. https://doi.org/10.1002/adma.202004240.

S2. Chen, G., Kang, Y., Yang, H., Zhang, M., Yang, J., Lv, Z., Wu, Q., Lin, P., Yang, Y., and Zhao, J. (2023). Toward forty thousand-cycle aqueous zinc-iodine battery: simultaneously inhibiting polyiodides shuttle and stabilizing zinc anode through a suspension electrolyte. Adv. Funct. Mater. *33*, 2300656. https://doi.org/10.1002/adfm.202300656.

S3. Wang, S., Huang, Z., Tang, B., Li, X., Zhao, X., Chen, Z., Zhi, C., and Rogach, A.L. (2023). Conversion-type organic-inorganic tin-based perovskite cathodes for durable aqueous zinc-iodine batteries. Adv. Energy Mater. *13*, 2300922. https://doi.org/10.1002/aenm.202300922.

S4. Ji, Y., Xie, J., Shen, Z., Liu, Y., Wen, Z., Luo, L., and Hong, G. (2023). Advanced zinc-iodine batteries with ultrahigh capacity and superior rate performance based on reduced graphene oxide and water-in-salt electrolyte. Adv. Funct. Mater. *33*, 2210043. https://doi.org/10.1002/adfm.202210043.

S5. Wu, J., Yang, J.-L., Zhang, B., and Fan, H.J. (2024). Immobilizing polyiodides with expanded Zn^{2+} channels for high-rate practical zinc-iodine battery. Adv. Energy Mater. *14*, 2302738. https://doi.org/10.1002/aenm.202302738.

S6. Xiao, T., Yang, J.-L., Zhang, B., Wu, J., Li, J., Mai, W., and Fan, H.J. (2024). All-

round ionic liquids for shuttle-free zinc-iodine battery. Angew. Chem. Int. Ed. *63*, e202318470. https://doi.org/10.1002/anie.202318470.

S7. He, J., Hong, H., Hu, S., Zhao, X., Qu, G., Zeng, L., and Li, H. (2024). Chemisorption effect enables high-loading zinc-iodine batteries. Nano Energy *119*, 109096. https://doi.org/10.1016/j.nanoen.2023.109096.

S8. Dang, H.X., Sellathurai, A.J., and Barz, D.P.J. (2023). An ion exchange membrane-free, ultrastable zinc-iodine battery enabled by functionalized graphene electrodes. Energy Storage Mater. *55*, 680–690. https://doi.org/10.1016/j.ensm.2022.12.033.

S9. Chen, Z., Wang, F., Ma, R., Jiao, W., Li, D., Du, A., Yan, Z., Yin, T., Yin, X., Li, Q., et al. (2024). Molecular catalysis enables fast polyiodide conversion for exceptionally long-life zinc-iodine batteries. ACS Energy Lett. *9*, 2858–2866. https://doi.org/10.1021/acsenergylett.4c00992.

S10. Guo, X., Xu, H., Tang, Y., Yang, Z., Dou, F., Li, W., Li, Q., and Pang, H. (2024). Confining iodine into metal-organic framework derived metal-nitrogen-carbon for long-life aqueous zinc-iodine batteries. Adv. Mater. *36*, 2408317. https://doi.org/10.1002/adma.202408317.
